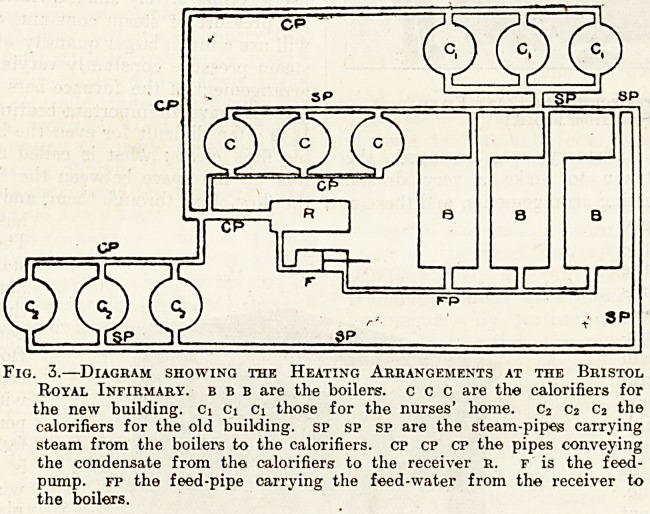# Bristol Royal Infirmary

**Published:** 1916-02-05

**Authors:** 


### THE HEATING OF HOSPITALS.*

**NOTE.—In a series of articles the writer proposes to describe the different systems of heating that are employed in hospitals. He will be pleased to answer any questions through "The Hospital," bearing upon the subject of heating or ventilation. Each system will be illustrated and described as exemplified at some hospital where it has been applied.**

### VII. Bristol Royal Infirmary.

### OPEN AIR-HEATING FIRES, ASSISTED BY ACCELERATED HOT-WATER RADIATORS.

In Bristol, as in the North, economy is the striking feature of all heating apparatus that has been put in during recent years. When the new build-

Fig. 1.—Water-softening Plant Fixed. (Seen from above.)

ing of the Royal Infirmary was arranged, the opportunity was taken to make a very drastic alteration in the heating arrangements, and thereby to introduce very considerable economies. Under the new arrangements there are three heating stations, all supplied with steam from one central battery of boilers; one for the old building, one for the new building, and one for the nurses' home. The arrangement enabled nine coal-fired steam boilers and three gas boilers to be done away with, in addition to providing all the heat required for the new building and the nurses' home. The boiler room is below the level of the road, under the new building, and a bunker sufficient for holding 110 tons occupies a space under the road, the coal being delivered to the bunker through the usual traps closed by iron plates in the yard above. The coal is allowed to fall from the bunker on to the boiler-room floor, behind iron screens fixed opposite each boiler, as it is required.

There are three Lancashire boilers, each 28 feet long by 7 feet in diameter, made to work up to a pressure of 80 lb. per square inch. One boiler is sufficient in summer, two are required in winter, and one is kept as a spare. The boiler furnaces are fitted with rocking bars in place of the usual fixed bars. These enable very much cleaner fires to be kept while using the inferior qualities of coal, and are claimed to be economical. The furnaces of steam boilers, and their proper handling, have a very important bearing upon the economy possible in heating. It is proverbial that a good stoker will use a comparatively small quantity of coal and keep the pressure of steam constant, while a bad stoker will use a much larger quantity of coal and have his steam pressure constantly varying. The form and arrangement of the furnace bars and the quality of the coal have an important bearing upon the matter. It is often difficult for even the best stoker to keep his fires clean; what is called clinker forms, and fills up the space between the fire bars, checking the flow of air through them, and reducing the combustion of the fuel. The central heating station for the new building is close to the boiler room. It contains a large Boby water softener, capable of dealing with 1,000 gallons per hour, shown in fig. 1. Bristol water is hard, hence the water-softening plant. Town's water is delivered to the water softener, and is forced by a Rees Roturbo electrically driven pump to an 800-gallon tank on the roof, after being softened. There are three calorifiers in each heating station; one for heating the water for the radiators, one for the hot-water supply, and the third to take the place of either. The calorifiers are interconnected so that each can be used for either purpose. The water from the

Fig. 2.—Air-heating Coal-burning Stoves in Use in the New Building of Bristol Royal Infirmary.

*Previous articles appeared on July 31, September 11, October 2 and 16, November 13, 1915, and January 15, 1916.

tank in the roof of the new building flows by gravity to the calorifiers to make up for the water consumed for domestic purposes and for the waste in the radiator system. Both the hot-water supply and the radiator system in the new building are "accelerated" by electrically driven Rees Roturbo pumps. The steam space of each calorifier is connected to a steam trap which carries the condensate and any steam which comes over with it to a receiver in the heating station of the new building, from which it is pumped into the boiler by a Weir boiler-feed pump, arranged for dealing with hot water. The receiver in the heating station of the new building receives all the condensed water from all parts of the three buildings, except that from the laundry engine. The water required for the boilers, in addition to that obtained from condensed steam, is also taken from the tank on the roof, and is heated by a small feed-water heater fixed in the central heating station, the steam employed to heat the water being the exhaust from the boiler feed pump. There are steam heating appliances in different parts of the building, steam sterilisers and various other apparatus; the water formed by condensation in them is carried to the receiver in the central heating station. The calorifiers for heating the old building are 350 feet from the boilers, and those in the nurses' home are 500 feet away

The laundry, which is 620 feet from the boilers beyond the old building, is supplied with steam by a 3-inch pipe running in an old subway believed to have been used by the monks.

Fig. 3.—Diagram showing the Heating Arrangements at the Bristol Royal Infirmary. b b b are the boilers. c c c are the calorifiers for the new building. $C_1$ $C_1$ $C_1$ those for the nurses' home. $C_2$ $C_2$ $C_2$ the calorifiers for the old building. sp sp sp are the steam-pipes carrying steam from the boilers to the calorifiers. cp cp cp the pipes conveying the condensate from the calorifiers to the receiver r. f is the feed-pump. fp the feed-pipe carrying the feed-water from the receiver to the boilers.

### The Heating and Ventilation of the Wards.

Heating of the wards in the new building is accomplished principally by two pairs of air-heating stoves placed on the middle line of each ward, back to back as usual, the stoves being spaced so that the heat is equally distributed over the ward. Fig. 2 shows the stoves used, made by Messrs. Shorland and Brother, of Manchester.

The supply of air to be heated by each stove before passing into the ward is regulated by dampers controlled from the sides of the stoves. The hot flue gases from the fires are taken out, through ducts, to chimneys running up outside of the building, and all the ducts are swept from boxes provided near the bottoms of the chimneys in the yard outside. In addition to the coal-burning stoves, 3-inch hot-water pipes run round the wards, and there are two hot-water radiators at one end of each ward. The writer understands that the heating of the wards by the above is quite sufficient, and that it is not often necessary to light more than one of each pair of the stoves. Ventilation is absolutely natural. The radiators are regulated by two valves, one the usual wheel screw-down valve and the other a locked screw-down valve. The locked valve is set for the flow of a certain maximum quantity of water when the wheel valve is wide open; hence by moving the wheel valve up or down the flow of water and the heat delivered by the radiator can be regulated within the limit provided by the screw-down valve.

An expansion joint is fixed in the pipes leading to each ward; this is found better than providing large bends. The temperature of the water in the heating pipes and radiators is kept at from 190° F. downwards, according to the temperature of the atmosphere outside. The corridors are heated very much in the same manner as the wards, partly by 3-inch pipes which run along all of them, and partly by radiators which are fixed at intervals, and connected to the pipes. There are nearly 300 radiators in all the buildings. The chapel, sitting rooms, etc., are heated in the same manner. The subway connecting the old building with the new, running under the road which divides the two buildings, is heated by hot-water pipes running along one wall.

### The Heating of the Old Building.

The old building is heated partly by open fires and partly by hot-water radiators. The radiators and the hot-water supply are worked on the thermo-syphon system. An electrically driven Rees Roturbo circulating pump was put in, but as the pipes in the old building were not changed, and they were so much larger than those in the new building, acceleration was found not to be required. The heating station for the old building is in a sub-basement, and it contains an evaporator in addition to the three calorifiers. A sirocco fan has been fixed to cool the atmosphere in the heating station. The condensate from the calorifiers is pumped back to the receiver in the heating station in the new building.

Fig. 3 shows diagramatically the arrangement of the boilers and the three heating stations.

## Figures and Tables

**Fig. 1. f1:**
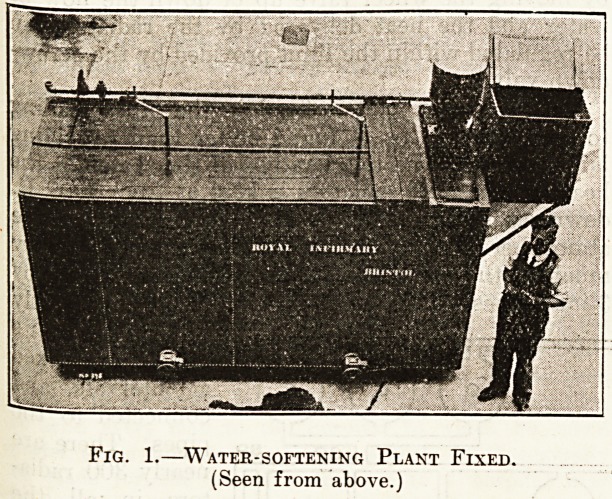


**Fig. 2. f2:**
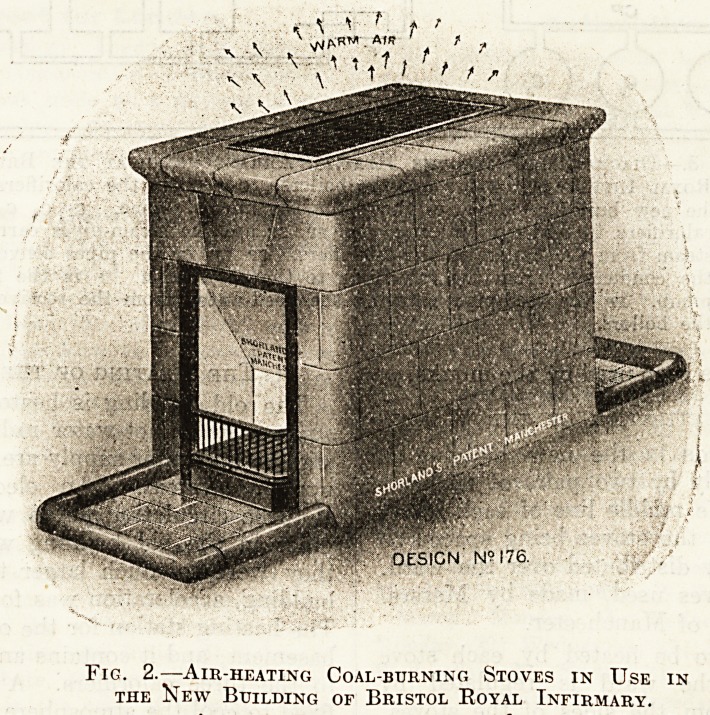


**Fig. 3. f3:**